# A web-database of mammalian morphology and a reanalysis of placental phylogeny

**DOI:** 10.1186/1471-2148-7-108

**Published:** 2007-07-03

**Authors:** Robert J Asher

**Affiliations:** 1 Museum of Zoology, University of Cambridge, Downing Street, Cambridge, CB2 3EJ, UK

## Abstract

**Background:**

Recent publications concerning the interordinal phylogeny of placental mammals have converged on a common signal, consisting of four major radiations with some ambiguity regarding the placental root. The DNA data with which these relationships have been reconstructed are easily accessible from public databases; access to morphological characters is much more difficult. Here, I present a graphical web-database of morphological characters focusing on placental mammals, in tandem with a combined-data phylogenetic analysis of placental mammal phylogeny.

**Results:**

The results reinforce the growing consensus regarding the extant placental mammal clades of Afrotheria, Xenarthra, Euarchontoglires, and Laurasiatheria. Unweighted parsimony applied to all DNA sequences and insertion-deletion (indel) characters of extant taxa alone support a placental root at murid rodents; combined with morphology this shifts to Afrotheria. Bayesian analyses of morphology, indels, and DNA support both a basal position for Afrotheria and the position of Cretaceous eutherians outside of crown Placentalia. Depending on treatment of third codon positions, the affinity of several fossils (*Leptictis*,*Paleoparadoxia*, *Plesiorycteropus *and *Zalambdalestes*) vary, highlighting the potential effect of sequence data on fossils for which such data are missing.

**Conclusion:**

The combined dataset supports the location of the placental mammal root at Afrotheria or Xenarthra, not at *Erinaceus *or rodents. Even a small morphological dataset can have a marked influence on the location of the root in a combined-data analysis. Additional morphological data are desirable to better reconstruct the position of several fossil taxa; and the graphic-rich, web-based morphology data matrix presented here will make it easier to incorporate more taxa into a larger data matrix.

## Background

Cladistic phylogeny reconstruction of mammals has its roots in publications by Malcolm McKenna [1] and was more explicitly algorithmic in the 1980s [2,3]. In the latter publications, discrete characters were analysed with an explicit optimality criterion, and were in principle observable by anyone with access to relevant material, in order to make specific, testable hypotheses regarding mammalian interrelationships. In retrospect, debate about mammalian interrelationships following these publications moved away from competing authoritarian statements on how mammalian groups are interrelated and towards a more focused discussion of the actual characters upon which such interrelationships are hypothesized [e.g., [4]].

Objections to algorithmic approaches to phylogeny reconstruction, particularly regarding its practice among morphologists [e.g., [5]], have occasionally noted the uninformative and/or low quality of character descriptions. Individual investigators are not necessarily to fault for the format in which their character lists are published, as editorial standards for such information vary widely, not to mention the capacity of different journals to publish graphic and/or textual appendices. Nevertheless, calls for the improvement of standards by which morphological character data are published, and by which they are selected for inclusion in a given study, have been made [e.g., [6]].

Web-based databanks offer an ideal means by which the information content of anatomical character sets can be maximized. Initiatives such as: digimorph [36], morphobank.org [37] and morphbank.net [38] have for several years taken advantage of this medium [7] , and have made it easier for investigators to evaluate morphological data with the ultimate goal of better understanding character evolution and phylogeny. However, as of this writing, a databank focusing on the skeletal anatomy of placental mammals is still lacking.

### Hypotheses of placental mammal phylogeny

A widely cited dataset consisting primarily of nuclear DNA sequences [8,9] has been interpreted to contain an unambiguous signal dividing placental mammals into four main clades: Xenarthra (armadillos, sloths, and anteaters), Afrotheria (sea cows, elephants, hyraxes, elephant shrews, aardvarks, tenrecs, and golden moles), Euarchontoglires (primates, tree shrews, colugos, rodents, and lagomorphs), and Laurasiatheria (lipotyphlans, bats, carnivorans, pangolins, perissodactyls, whales, and artiodactyls), with a root separating Afrotheria from the remaining placental mammals. Studies of mtDNA that include both coding and non-coding sequences [10], as well as the longest concatenation of nuclear DNA to date [11], with ca. 200,000 aligned nucleotides for 18 terminal taxa, support this topology.

Other DNA datasets, including analyses of rare molecular features such as the presence/absence of retroposons [12] and sequence analysis of LINEs [13], provide independent support for the same unrooted topology, but disagree on the location of the root. This falls either at Atlantogenata (Afrotheria+Xenarthra) [13,14], Xenarthra [12], or Glires (Rodentia+Lagomorpha) [15]. Earlier analyses of mitochondrial protein-coding genes [16] and of a combined morphology+DNA dataset [17] have also supported a basal (and often paraphyletic) position of rodents, although in [16] erinaceids were located at the placental root, adjacent to murid rodents. The most recent molecular phylogenetic analyses of placental mammals support a relatively basal position of afrotherians and xenarthrans (except for [15]), and a monophyletic Rodentia and Glires [18], but the precise identity of the basal-most placental taxon remains elusive.

Palaeontological work continues to yield fossil mammals that are relevant to debates on mammalian phylogeny and the placental root [19-21]. Some have argued that certain Cretaceous eutherians comprise the sister taxon to Glires [22]. If Cretaceous eutherian lineages could be definitively linked with modern rodents and lagomorphs, this could be interpreted to support to the hypothesis of Glires basal within Placentalia [15]. However, the most taxon- and character-rich phylogenetic analyses including Cretaceous eutherians [20,21] do not support their placement within crown Placentalia, nor are they unanimous in identifying a basal-most crown placental clade.

In this paper, I present an image-rich, morphological character-database focusing on placental mammals, in tandem with a reanalysis of morphological and sequence data that bear on placental mammal phylogeny. The morphological character list is based on [17], which was in turn based on the work of many other publications, as cited therein. I combine these morphological data with the DNA sequence dataset (19 nuclear and 3 mitochondrial genes) of [9], and for the first time include information on 221 indels from their DNA sequence alignment. I apply a number of corrections to both the sequence- and morphological data sets; and using both maximum parsimony (MP) and a Bayesian algorithm, I investigate the support of these data for the aforementioned hypotheses on mammalian interrelationships and the placental root.

## Results and Discussion

**Figure 1:**
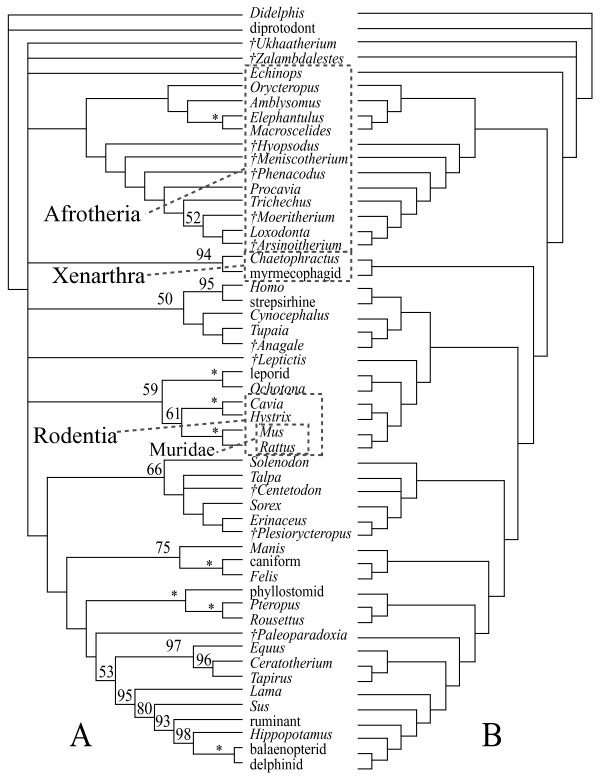
**Optimal MP topologies, all data**. Strict (A) and Adams (B) consensuses of 4 trees (49750 steps) resulting from combined morphology-DNA-indel dataset, all changes treated equally. Numbers indicate bootstrap support values (only reported above 50); asterisks indicate support of 100. Daggers indicate extinct taxa.

**Figure 2:**
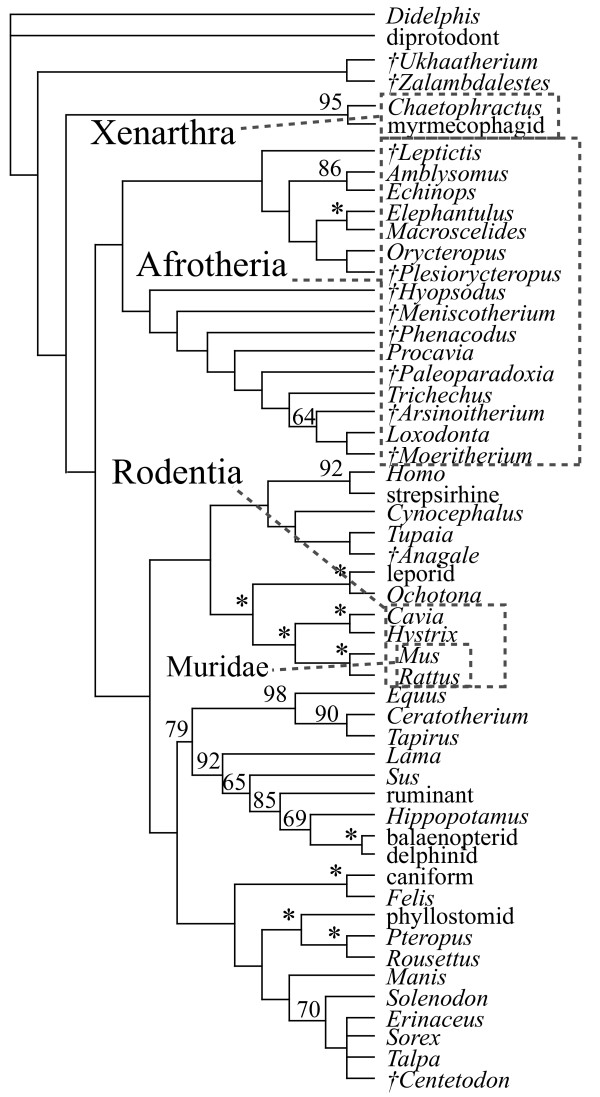
**Optimal MP topologies, third positions removed**. Strict consensus of 4 trees (27858 steps) resulting from combined morphology-DNA-indel dataset, excluding third positions from protein-coding genes. Numbers indicate bootstrap support values (only reported above 50); asterisks indicate support of 100. Daggers indicate extinct taxa.

**Figure 3:**
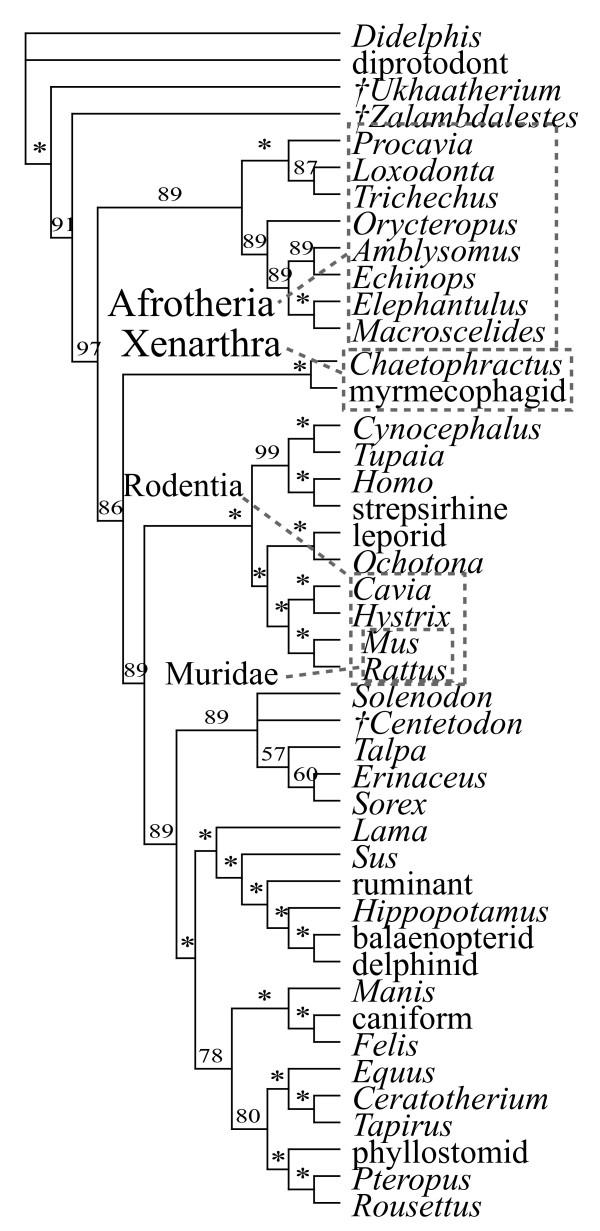
**Bayesian tree**. Majority rule consensus of 15500 trees (1.6 million generations, sampled every 100, first 500 discarded as "burn-in") generated by MrBayes 3.1 [33]. Numbers indicate Bayesian posterior probability values; asterisks indicate support of 100. Daggers indicate extinct taxa.

The majority of the combined DNA-morphology analyses support the clades Afrotheria, Xenarthra, Euarchontoglires, and Laurasiatheria, as well as the placement of the Tertiary insectivoran-grade mammal *Centetodon *within Lipotyphla and the two Cretaceous eutherians (*Ukhaatherium *and *Zalambdalestes*) outside of Placentalia (Figs. 1, 2, 3). Using MP, the position of the placental root varies. With all data and gaps included and weighted equally (Fig. 1), or with third position transitions removed, it is at the Malagasy lesser hedgehog-tenrec *Echinops*, within a paraphyletic Afrotheria. A strict consensus in each case leaves the placental base unresolved (Fig. 1A) due to the variable position of *Zalambdalestes*. With third positions of protein-coding genes removed, it is at Xenarthra followed by Afrotheria with Cretaceous taxa outside of crown Placentalia (Fig. 2). Results from the Bayesian analysis using either living taxa and sequence data alone, or including three fossils (*Zalambdalestes*, *Ukhaatherium*, and *Centetodon*) plus morphology (Fig. 3), places the placental root at Afrotheria followed by Xenarthra. When included, Cretaceous taxa are again reconstructed outside of crown Placentalia.

**Figure 4:**
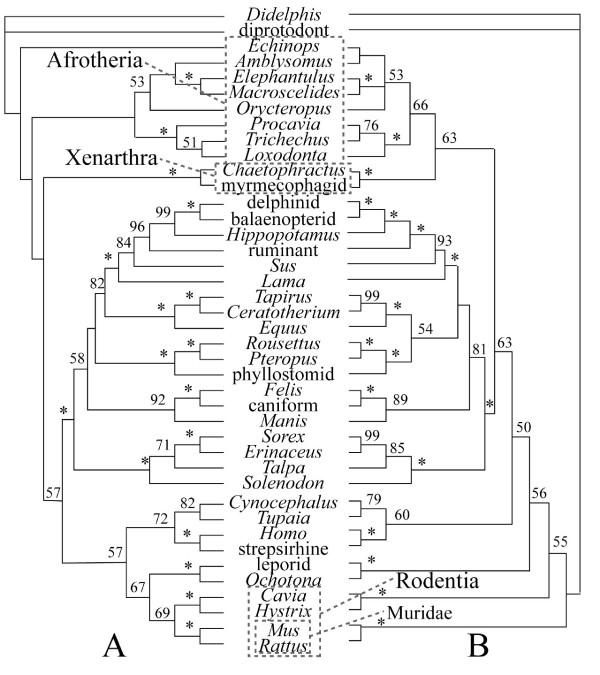
**Optimal MP topologies for Recent taxa alone**. The analysis of morphology-DNA-indels (A) yields a single tree of 49588 steps with the placental root within Afrotheria. Using DNA-indels alone (B) yields two trees at 48530 steps with placental root at murid rodents. Numbers indicate bootstrap support values (only reported above 50); asterisks indicate support of 100.

Interestingly, MP applied only to extant taxa with all DNA characters, but without morphology, yields a placental tree rooted on murid rodents (Fig. 4B). Inclusion of morphology changes this signal to favour a root within Afrotheria, at the Malagasy tenrec *Echinops *(Fig. 4A). Removal of third positions favours a placental root at Xenarthra (Fig. 2) with or without morphological data. As evident by comparing Figs. 1B and 4A, exclusion of the 12 fossil taxa in the equally weighted MP analysis does not shift the root away from the afrotherian *Echinops*.

Table 1 summarizes the results of Templeton and Winning Sites tests using PAUP 4.0b10 [23] evaluating competing hypotheses on the location of the placental root. Using MP applied to the combined dataset, and regardless of the treatment of third positions, the hypotheses of Glires or *Erinaceus *basal are rejected. With third coding positions excluded, these tests yield p-values close to but not consistently below 0.05 for both Atlantogenata and Muridae at the placental root. With all DNA-indel-morphology characters included, Atlantogenata is rejected and Muridae is not. Monophyletic, basal Afrotheria or Xenarthra is not rejected in any case (Table 1).

**Table 1:** Templeton and Winning Sites tests of alternative topologies, varying placement of the placental root, based on MP with all DNA, indels, and morphology (1–7) and excluding DNA 3rd positions (8–13). Trees 1 and 8 are unconstrained. Competing topologies 2–7 and 9–13 were generated by analysis of the combined dataset with MP constrained to agree with backbone topology supporting Afrotheria [9], Atlantogenata [14], Xenarthra [12], Muridae (Fig. 4B), Erinaceus [16], and Glires [15]. Asterisks indicate rejection of no difference between optimal and competing topology at alpha 0.05.

		Templeton				Winning-sites	
Taxon at root	Length	Rank sums	N	z	P	counts	P

1. MP-best equal weighting (Echinops, paraphyletic Afrotheria)	49750	(best)					
2. Afrotheria	49790	163202.5 -147663.5	788	-1.3911	0.1642	411 -377	0.2398
3. Atlantogenata	49826	205640.0 -173245.0	870	-2.4941	0.0126*	470 -400	0.0193*
4. Xenarthra	49777	137166.0 -127462.0	727	-0.9796	0.3273	372 -355	0.5529
5. Muridae	49761	415894.0 -410361.0	1285	-0.2359	0.8135	649 -636	0.7378
6. Erinaceus	50166	1116885.0 -752326.0	1933	-8.2408	< 0.0001*	1154 -779	< 0.0001*
7. Glires	49876	436500.0 -361716.0	1263	-3.2480	0.0012*	694 -569	0.0005*
8. MP-best 3rd positions excluded (Xenarthra)	27858	(best)					
9. Afrotheria	27894	25156.0 -19994.0	300	-1.9270	0.0540	164 -136	0.1190
10. Atlantogenata	27904	27723.5 -21104.5	312	-2.3113	0.0208*	173 -139	0.0617
11. Muridae	27915	76399.5 -62728.5	527	-2.2030	0.0276*	286 -241	0.0553
12. Erinaceus	28137	236909.0 -131602.0	858	-7.9545	< 0.0001*	551 -307	< 0.0001*
13. Glires	27951	33510.0 -18816.0	323	-4.9874	< 0.0001*	208 -115	< 0.0001*

The position of the placental root influences the optimization of morphological characters throughout the placental tree. However, some morphological characters optimize at the root of Placentalia under a number of hypotheses. With either Afrotheria, Xenarthra, Atlantogenata, Glires, Muridae, or *Erinaceus *at the placental base, three morphological character states optimize as placental synapomorphies: #39-1 (single hypoglossal foramen), #48-0 (foramen rotundum confluent with sphenorbital fissure), and #159-1 (epipubic bones absent). With either Afrotheria or Atlantogenata basal, two additional morphological synapomorphies for Placentalia optimize unambiguously: #11-0 (presence of a sulcus for the internal carotid artery on the promontorium of the petrosal) and #105-1 (prominent lingual cusp on upper P3). A paraphyletic Rodentia at or near the placental base (following [16] or Fig. 4B) greatly increases the number of morphological characters that show unambiguous change on the branch leading to crown Placentalia, and requires significantly more homoplasy among morphological characters than the other hypotheses of rooting.

The placement of several fossils, namely *Leptictis*,*Paleoparadoxia*, *Plesiorycteropus *and *Zalambdalestes*, remains ambiguous in this study. However, when resolved, the latter taxon falls outside of crown Placentalia (Figs. 2, 3); this result has also been supported by other, independent datasets [20,21]. In the current study, the treatment of DNA third positions influences the topology of several fossils, a result that may appear counterintuitive since all DNA data are missing for these fossils. Nevertheless, this is a straightforward result based on the altered optimizations of morphological characters on those branches of the tree that are rearranged by addition of the sequence partition, which in turn can affect the influence of those characters on the placement of fossils [24].

## Conclusion

Compared to just a decade ago, there is now a broad level of agreement on the basic topology of the extant mammalian radiation [e.g., [8-14]]. Using a relatively large DNA-indel-morphology dataset based on [8,9,17], this study has made a number of changes to both molecular and morphological homology (see additional file 1), yet recovers the same basic pattern of living placental phylogeny (Figs. 1, 2, 3), dividing the unrooted tree into Afrotheria, Xenarthra, Euarchontoglires, and Laurasiatheria.

The same level of agreement cannot yet be said to exist for all fossil clades. In this study, *Ukhaatherium*, *Centetodon*, *Hyopsodus*, *Meniscotherium*, *Phenacodus*, *Arsinoitherium*, *Moeritherium*, and *Anagale *are placed with some consistency across analyses. The remaining four fossils (*Leptictis*, *Paleoparadoxia*, *Plesiorycteropus*, and *Zalambdalestes*) vary in their position depending on the analysis, indicating that at present the morphological data sampled here are not sufficient to reconstruct the phylogeny of these taxa. I concur with [25] that the current morphological sample could be expanded significantly. Nevertheless, this study demonstrates that even a small morphological dataset can influence a much larger body of DNA sequences. Here, morphology not only improves resolution in some clades that remain poorly resolved based on DNA sequences alone (e.g., favouring sea cow-elephant), but can also shift the placental root from Muridae to Afrotheria (Fig. 4). The combined data favour a placental root at either Afrotheria or Xenarthra (Table 1; Figs. 1, 2, 3). Both Atlantogenata and Muridae receive suggestively low p-values with third coding positions excluded; Glires and *Erinaceus *are the least favoured root-taxa among the alternatives tested with the present dataset.

The morphological web-database presented here will make it easier for researchers to incorporate these data into larger phylogenetic matrices that sample additional fossils. In the long term, such representations will be essential to reconstruct the morphology of the placental common ancestor. Towards this end, morphological character matrices should be easily accessible and understandable across institutions and generations of scientists; and they should build upon previous work in order to offer an ever-expanding character database. Many kinds of molecular data have enjoyed such accessibility for well over a decade. The relatively infrequent presentation of graphic character databases limits the utility and appreciation of morphological character matrices, a condition that in recent years has, fortunately, begun to change.

## Methods

### The Website

The 196 characters first described in [17] are available in web-format via the author's institutional website [26] and is archived on the BMC website [see additional file 1]. With few exceptions, images were photographed using museum collections in Berlin (ZMB), New York (AMNH), Washington DC (USNM), London (NHM), Pretoria (TM), and Cambridge (UMZC). Images and character descriptions were combined and exported as JPEG or GIF files using Adobe Photoshop and Illustrator. These were linked into HTML files using Mozilla Composer.

### Morphology matrix

The current web-matrix includes corrections to Appendices 1 and 2 of [17] [see additional file 1]. Among the typographical errors listed, only one had an effect on the analysis: character 41 of *Tapirus *("mastoid exposure in braincase") was inadvertently omitted from the printed Appendix 1 from [17]. It should have been listed as state "0" for *Tapirus *(mastoid exposed). With this correction, and using either PAUP [23] or NONA [27] under the analytical defaults of POY 2.7 [28] (e.g., polymorphisms treated as missing data), the morphological dataset published in appendix 1 of [17] yields the reported 4 trees at 1088 steps.

The terms "fenestra rotunda", "fenestra cochleae", and "round window" have been used interchangeably for the aperture in the ventrum of the petrosal pars cochlearis, leading into the cochlea, just posterior to the fenestra vestibularis (or oval window; see [29]). Asher et al. [17,24] had previously used the descriptor "rotundum" for this structure in characters 4 and 5, which should have been reserved for the distinct exit foramen for the maxillary division of the trigeminal nerve (as in primates, carnivorans, and marsupials). In order to avoid confusion between the fenestra "rotunda" (round window) and the foramen "rotundum" (exit foramen for V-2), text and images for characters 4–7 now use the term "fenestra cochleae" for this opening on the ventrum of the pars cochlearis, following [29].

Relative to the descriptions first published in [17], the text for several characters has been changed in order to better correspond to the specimens available for display on the website.

In addition to the typographical corrections summarized above, some of the coding decisions in [17] have also been changed [see additional file 1], which of course do influence the structure of the tree. Six of these were indicated in [24]; four additional improvements are identified here.

First, instead of identifying a separate character state for "glenoid poorly defined" for character #56 in *Manis*, this character is coded as in most other mammals: state 0, "glenoid even with petrosal." This increases consistency in how the fossil taxon *Plesiorycteropus *was coded, and reflects the actual position of the glenoid fossa for the mandible in a transverse plane near the petrosal bone, as opposed to the dorsally situated glenoid in, for example, chrysochlorids or caviomorph rodents.

Second, the lacrimal bone (character #71) in leporid skulls is not always well ossified to surrounding bones, and in some specimens it may fall out leaving an artefactual "fenestra" in the anterior orbit. This was incorrectly coded in [17,24] as a separate character state, "fenestra in anterior orbit." Here, this is recoded in the leporid terminal as "lacrimal foramen present."

Third, *Didelphis *possesses a distinct foramen rotundum (i.e., exit foramen for the maxillary [2nd] division of the trigeminal nerve, character #48), just posterior to the sphenorbital fissure [30,31]. The foramen rotundum was mistakenly coded as "confluent with sphenorbital fissure" in [2,17,24]. It is here corrected to state 1 ("distinct") to reflect the ossified, separate exit foramen for the maxillary division of the trigeminal nerve in this taxon.

Fourth, character #39 "condyloid foramina" should have been worded to specifically indicate the hypoglossal foramen, reflecting the usage of [31]. As summarized by [[32]: p. 175], the terms "condylar" or "condyloid" foramen have been used for this structure [2]. However, the descriptor "condylar" or "dorsal condylar" may also refer to small, nutrient foramina adjacent to the occipital condyle [[32]: p. 151]. Several taxa show multiple foramina that perforate the basioccipital anterior to the occipital condyle (e.g., *Didelphis*); others show a single, conspicuous hypoglossal foramen (e.g., *Pteropus*), and others lack a hypoglossal foramen (e.g., *Balaenoptera*). Asher et al. [17,24] had previously coded *Orycteropus, Sus*, and *Sorex *as lacking hypoglossal foramina; here, these codings are corrected to state 1 ("single") for the former two, and states 0 and 1 (polymorphic) for *Sorex*.

### DNA sequence and indel dataset

Sequences of the tyrosinase (TYR) gene in *Equus *(accession AF252540) were added to the alignment of [9]. In addition, several interruptions of the reading frame and placements of several indels were adjusted (see additional file 1), amounting to 34 alterations in presumed sequence homology. In addition, 221 insertion-deletion indel characters from protein-coding genes in this DNA dataset were incorporated into a new phylogenetic analysis using MP [23] and MrBayes [33]. Each indel character is coded as 0 (for gaps) or 1 (for insertions) and consists of one or more units of three contiguous gaps. Regardless of length, such occurrences were coded as a single, binary character, shared by two or more taxa when they show overlap. Elongate gaps that overlapped with multiple, smaller gaps were coded as a single event; i.e., when an elongate gap character in taxon A overlapped with multiple, smaller gap characters in taxa B and C, the smaller gap-characters were coded as inapplicable for taxon A and treated as missing data in the analysis, based on the method of "simple indel coding" [34]. The newly-aligned sequence dataset is available linked to additional file 1. Exclusion of sites identified as "alignment ambiguous" by [9] did not have a significant effect on the topologies reported here.

### Taxon sample

The choice of Recent taxa for inclusion in this dataset is based on maximizing the overlap of the morphological dataset with the 19 nuclear and 3 mitochondrial gene dataset used by [9]. This is the same sample used by [24], and is slightly smaller than that used by [17], including 41 extant and 12 extinct mammalian terminals. Not included are the sciurid, *Bradypus, Tadarida*, and *Vampyrum *sequences used by [9]; and a single terminal is used for the Caribbean lipotyphlan *Solenodon *(using sequence data for *Solenodon paradoxus*). Several terminal taxa are composites, listed here with suprageneric names, and are identified in table 1 of [24].

### Phylogenetic analysis parameters

Different schemes for weighting third positions codons in MP (excluded, transitions ignored, included) were explored. Sequence data for all fossils were coded as missing; all morphological character changes were treated as nonadditive (unordered). In all MP analyses, multistate characters were treated as polymorphic, indel characters embedded in the sequence data matrix were treated as missing data (but were represented in an additional character matrix), and tree searches using PAUP [23] were heuristic using at least 200 random addition replicates and TBR branch-swapping. Bootstrap values are based on at least 100 pseudoreplicates of a 3-replicate TBR random addition sequence.

Analyses with MrBayes [33] used the AIC as applied in MrModeltest [35], based on ML scores generated by PAUP [23], to determine the model of evolution for each genetic locus independently as well as for the combined nuclear and mitochondrial genes as two discrete partitions. In most cases this identified the GTR+G+I model as optimal (Table 2). Bayesian treebuilding was computationally intensive. Partitioning the data into units of nuclear (ca. 15KB) and mitochondrial (ca. 1.5KB) DNA, plus 221 indel characters, the former two with an independent GTR+G+I model and the latter with a restriction site model (as recommended in MrBayes documentation), and combining them with the datasets for morphology including fossil taxa, took 18 days for 2 million generations on a single mac G5 processor (2.5 GHz and 2.5 GB RAM) with MrBayes 3.1. This still did not yield convergence across two independent runs. Hence, Bayesian analyses included three of the 12 sampled fossils (plus all 41 Recent taxa), using just over 1.6 million generations in two independent runs, which yielded the same consensus of post-burnin topologies (Fig. 3).

**Table 2:** Models identified by the AIC in MrModeltest [35] as optimal for each gene and/or groups thereof.

**gene**	**model**	**gene**	**model**
a2ab	GTR+I+G	irbp	GTR+I+G
adora3	GTR+I+G	plcb4	GTR+I+G
adrb2	HKY+I+G	pnoc	GTR+I+G
app	GTR+G	rag1	GTR+I+G
atp7a	GTR+I+G	rag2	HKY+I+G
bdnf	GTR+G	tyr	SYR+I+G
brca1	GTR+I+G	vwf	GTR+I+G
cnr1	GTR+I+G	zfx	HKY+I+G
crem	GTR+I+G	mtRNA	GTR+I+G
edg1	GTR+I+G	nucDNA	GTR+I+G

Analysis of sequence data for the 41 extant terminals only, with three unlinked evolution models defined for nucDNA, mtRNA, and indels, yielded convergence for two independent runs after ca. 3 weeks of uninterrupted computing time for one million generations on a 2Ghz P4 desktop PC with 512MB RAM. Using 21 unlinked models of sequence evolution for each gene (Table 2) in two additional runs of one million generations each yielded the same post-burnin, majority rule consensus topology as the 3-model analysis. Based on manual inspection of likelihood scores, Bayesian analyses across these analyses reached stationarity after approximately 15K generations; burn-in was conservatively defined after 50K generations.

Statistical tests of competing topologies were carried out in PAUP 4.0b10 [23]. One of the four MPTs including all data with all changes equal (Fig. 1), and one of the four MPTs resulting from the analysis excluding third coding positions (Fig. 2), were compared with several alternatives (Table 1). Because of differences in taxon sample across studies concerning the root of Placentalia [e.g., [9,15,16]], these alternatives were constructed with the present dataset, using backbone-constraints derived from each study. For example, taxa from the present dataset sampled in common with [16] were constrained in PAUP to fit figure 1 from [16], which supported erinaceid insectivorans basal followed by murid rodents. One of the resulting MPTs was then compared to an unconstrained, optimal MPT using the present morphology-DNA-indel dataset under the assumptions given in Fig. 1 (equal weighting) and Fig. 2 (third positions excluded). The same procedure was followed for hypotheses supporting basal positions of Atlantogenata [14], Xenarthra [12], Afrotheria [9], Glires [15], and Muridae (Fig. 4B).

## Authors' contributions

RJA assembled the morphological and DNA sequence data matrices (the latter based on an alignment supplied by A. Roca and W. Murphy), designed the web-database, carried out the phylogenetic analyses, and wrote the manuscript. All authors read and approved the final manuscript.

## Supplementary Material

Additional File 1 Website data: The 196 characters first described in [17], along with the supplementary data indicated below, have been joined into a number of HTML files, all of which are linked to the "morphsite_bmc07.html" index page. The embedded links to the supporting files are functional, independent of PC or platform, provided that the directory structure remains intact (i.e., all files remain in a single folder, no subdirectories).
Nexus files: Datafiles containing the morphological, indel, and aligned DNA sequence data, linked from the "morphsite_bmc07.html" index page.
Table 1. Changes to the multiple sequence alignment of [9].
Table 2. Qualitative character-state corrections to the morphology matrix of [17].
Table 3. Summary of discrepancies between Appendices 1 and 2 of [17]. Click here for file

## References

[B1] McKenna MC, Luckett WP, Szalay FS (1975). Toward a phylogeny and classification of the Mammalia. Phylogeny of the Primates: a Multidisciplinary Approach.

[B2] Novacek M (1986). The Skull Of Leptictid Insectivorans And The Higher-Level Classification Of Eutherian Mammals. Bull Am Mus Nat Hist.

[B3] Rowe T (1988). Definition, diagnosis and origin of Mammalia. Journal of Vertebrate Paleontology.

[B4] Gaudin TJ, Wible JR, Hopson JA, Turnbull WD (1996). Reexamination of the morphological evidence for the Cohort Epitheria (Mammalia, Eutheria). Journal of Mammalian Evolution.

[B5] Cartmill M (1981). Hypothesis testing and phylogenetic reconstruction. Z Zool Syst Evol.

[B6] Wiens JJ (2000). Phylogenetic Analysis of Morphological Data.

[B7] Thacker PD (2003). Morphology: The shape of things to come. Bioscience.

[B8] Murphy WJ, Eizirik E, O'Brien SJ, Madsen O, Scally M, Douady CJ, Teeling E, Ryder OA, Stanhope MJ, de Jong WW, Springer MS (2001). Resolution of the early placental mammal radiation using Bayesian phylogenetics. Science.

[B9] Roca AL, Bar-Gal GK, Eizirik E, Helgen KM, Maria R, Springer MS, O'Brien SJ, Murphy WJ (2004). Mesozoic origin for West Indian insectivores. Nature.

[B10] Kjer KM, Honeycutt RL (2007). Site specific rates of mitochondrial genomes and the phylogeny of Eutheria. BMC Evolutionary Biology.

[B11] Nikolaev S, Montoya-Burgos JI, Margulies EH, Rougemont J, Nyffeler B, Antonarakis SE, NISC Comparative Sequencing Program (2007). Early History of Mammals Is Elucidated with the ENCODE Multiple Species Sequencing Data. PLoS Genet.

[B12] Kriegs JO, Churakov G, Kiefmann M, Jordan U, Brosius J, Schmitz J (2006). Retroposed elements as archives for the evolutionary history of placental mammals. PLoS Biol.

[B13] Waters PD, Dobigny G, Waddell PJ, Robinson TJ (2007). Evolutionary History of LINE-1 in the Major Clades of Placental Mammals. PLoS ONE.

[B14] Murphy WJ, Pringle TH, Crider TA, Springer MS, Miller W (2007). Using genomic data to unravel the root of the placental mammal phylogeny. Genome Research.

[B15] Kullberg M, Nilsson MA, Arnason U, Harley EH, Janke A (2006). Housekeeping genes for phylogenetic analysis of eutherian relationships. Mol Biol Evol.

[B16] Arnason U, Adegoke JA, Bodin K, Born EW, Esa YB, Gullberg A, Nilsson M, Short RV, Xu X, Janke A (2002). Mammalian mitogenomic relationships and the root of the eutherian tree. Proc Natl Acad Sci USA.

[B17] Asher R, Novacek MJ, Geisler J (2003). Relationships of Endemic African Mammals and their Fossil Relatives based on Morphological and Molecular Evidence. Journal of Mammalian Evolution.

[B18] Kriegs JO, Churakov G, Jurka J, Brosius J, Schmitz J (2007). Evolutionary history of 7SL RNA-derived SINEs in Supraprimates. Trends in Genetics.

[B19] Ji Q, Luo ZX, Yuan CX, Wible JR, Zhang JP, Georgi JA (2002). The earliest known eutherian mammal. Nature.

[B20] Wible JW, Rougier GW, Novacek MJ, Asher RJ (2007). Cretaceous eutherians and Laurasian origin for placental mammals near the K/T Boundary. Nature.

[B21] Asher RJ, Meng J, Wible JR, McKenna MC, Rougier GW, Dashzeveg D, Novacek MJ (2005). Stem Lagomorpha and the antiquity of Glires. Science.

[B22] Archibald JD, Averianov AO, Ekdale EG (2001). Late Cretaceous relatives of rabbits, rodents, and other extant eutherian mammals. Nature.

[B23] Swofford DL (2002). PAUP* Phylogenetic Analysis Using Parsimony (*and Other Methods) Version 4.

[B24] Asher RJ, Emry RJ, McKenna MC (2005). New material of *Centetodon *(Mammalia, Lipotyphla) and the importance of (missing) DNA sequences in systematic paleontology. Journal of Vertebrate Paleontology.

[B25] Robinson TJ, Seiffert ER (2004). Afrotherian origins and interrelationships: new views and future prospects. Curr Top Dev Biol.

[B26] Morphological web database. http://people.pwf.cam.ac.uk/rja58/database/morphsite_bmc07.html.

[B27] Goloboff P (1993). NONA version 1.9 computer program. http://www.cladistics.com.

[B28] Wheeler WC, Gladstein D, DeLaet J (2002). POY direct optimzation computer program. http://research.amnh.org/scicomp/projects/poy.php.

[B29] Wible JR, Novacek MJ, Rougier GW (2004). New data on the skull and dentition in the Mongolian Late Cretaceous eutherian mammal *Zalambdalestes*. Bulletin Of The American Museum Of Natural History.

[B30] Gregory WK (1910). The Orders of Mammals. Bulletin of the American Museum of Natural History.

[B31] Wible JR (2003). On the cranial osteology of the short-tailed opossum Monodelphis brevicaudata (Didelphidae, Marsupialia). Annals of Carnegie Museum.

[B32] Wible JR, Gaudin TJ (2004). On the cranial osteology of the yellow armadillo Euphractus sexcintus (Dasypodidae, Xenarthra, Placentalia). Annals Of Carnegie Museum.

[B33] Ronquist F, Huelsenbeck JP (2003). MrBayes 3: Bayesian phylogenetic inference under mixed models. Bioinformatics.

[B34] Simmons MP, Ochoterena H (2000). Gaps as characters in sequence-based phylogenetic analyses. Syst Biol.

[B35] Nylander JAA (2004). MrModeltest v2. Program distributed by the author.

[B36] Digimorph database. http://www.digimorph.org.

[B37] Morphobank database. http://www.morphobank.org.

[B38] Morphbank database. http://www.morphbank.net.

